# My Story and Me: protocol for a feasibility study of a personalised public mental health intervention for young women aged 14–18 years

**DOI:** 10.1136/bmjopen-2025-115245

**Published:** 2026-05-06

**Authors:** Angelika Labno, Kathryn M. Abel, Hil Aked, Kamaldeep Bhui, Helen Brooks, Maddison Crease, Jessica Deighton, Chloe Edridge, Peter Fonagy, Eleanor Grace, Margaret Heslin, Suzet Tanya Leyera, Kirsty Nisbet, Adetola Obateru, Sam Norton, Giulia Ravaccia, Emily Stapley, Julian Edbrooke-Childs

**Affiliations:** 1 Anna Freud Centre, London, UK; 2 University College London, London, England, UK; 3 The University of Manchester, Manchester, England, UK; 4 Gendered Intelligence, London, UK; 5 University of Oxford, Oxford, UK; 6 Manchester Metropolitan University, Manchester, England, UK; 7 King’s College London, London, England, UK; 8 University of Dundee, Dundee, Scotland, UK; 9 Centre for Mental Health, London, England, UK

**Keywords:** MENTAL HEALTH, PUBLIC HEALTH, Adolescents, Feasibility Studies, Implementation Science

## Abstract

**Abstract:**

**Introduction:**

Rates of mental health difficulties among girls and young women in the UK have risen sharply, and disproportionately so for those from marginalised groups. My Story and Me is a new digital public mental health intervention that uses storytelling to reduce stigma, increase awareness and support early help-seeking among girls and young women aged 14–18. The feasibility study aims to determine the acceptability of the intervention and future full trial, including assessing optimal settings and meaningful changes in the primary outcome measure (anxiety and depression).

**Methods and analysis:**

This is an 18-month mixed-methods, uncontrolled feasibility study conducted in secondary schools, further education colleges and community organisations across the UK. We will recruit 120–180 participants. Quantitative data will be collected at baseline and 7-month follow-up. The primary outcomes are anxiety and depression, and secondary outcomes are social support, mentalising, stigma, quality of life, loneliness, empowerment, intervention acceptability, resource use and randomisation acceptability. Platform-level engagement data will assess adherence and fidelity. Qualitative interviews with young women and staff will explore acceptability, feasibility, mechanisms of change and views on trial procedures, including randomisation in a future full trial. Analysis will be descriptive and exploratory, including comparisons across settings and priority groups (LGBTQIA+, neurodivergent and those experiencing digital poverty). A framework and reflexive thematic analysis approach will be used for qualitative data. Prespecified progression criteria will inform decisions about advancing to a full cluster randomised trial.

**Ethics and dissemination:**

The University College London Research Ethics Committee (0692) has approved the My Story and Me protocol. Interested participants will be required to complete an expression of interest and consent form to take part in the study, and young people under 16 years old will be required to obtain parent/carer informed consent. Results will be disseminated through peer-reviewed publications, lived experience summaries, a policy briefing and academic conference presentations.

**Trial registration number:**

ISRCTN12191423.

**STRENGTHS AND LIMITATIONS OF THIS STUDY**

- This study will provide feasibility data for a new digital storytelling intervention designed specifically for girls and young women, including those from marginalised groups.
- Trialling the intervention in three different settings and using site logs will enable detailed evaluation of acceptability, feasibility and setting-specific implementation facilitators and barriers.
- Youth participation is embedded throughout the study design, including a peer researcher and a Young People’s Advisory Group shaping the intervention, measures and interpretation of results.
- The single-arm study design limits the ability to fully examine the acceptability of randomisation.
- The number of outcomes measured in this study may increase the risk of young people disengaging during data collection, resulting in incomplete or missing data.

## Introduction

 Mental health difficulties for young women are increasing. One in four young women, aged 17–19 years, experiences mental health difficulties; a rise from one in eight in 2017,^1^ and twice as many young women aged 17–25 experience mental health difficulties compared with young men. From early adolescence, young women have higher levels of depression and anxiety and lower levels of well-being than young men, and this gap widens through mid-adolescence.^2^ The WHO identifies gender as being a structural determinant of mental health.^3^ It is known that, throughout the life-course, women are more likely to be disadvantaged by societal structures.^4 5^ This includes socioeconomic deprivation, childbearing and caring responsibilities, discrimination, harassment, trauma and abuse.^6 7^ In particular, recent evidence from schools and colleges shows highly concerning levels of sexual harassment, affecting up to 90% of young women.^8^ The mental health of young women and marginalised groups has also been disproportionately affected by the impact of the COVID-19 pandemic.^9 10^

In general, young people from marginalised groups experience higher levels of mental health symptoms^11^ and greater inequalities in receiving mental health support.^12^ However, exploration of trajectories of mental health for black, minoritised and multiply disadvantaged groups has been limited. Young people and adults from minoritised ethnic groups are more likely to receive mental healthcare through compulsory routes than voluntary ones, compared with those from white British groups.^12 13^ Research also indicates that children and young people from Asian and mixed-race backgrounds are less likely to experience improvement in mental health difficulties over the course of receiving support from specialist child and adolescent mental health services (CAMHS), compared with those from white British groups.^14^ Furthermore, young women and men from LGBTQIA+ groups (lesbian, gay, bisexual, transgender, queer or questioning, intersexual, asexual and other communities) are at higher risk of depression, anxiety and suicide.^15 16^ Neurodivergent needs for young women are often unrecognised or mislabelled, delaying access to effective support.^17^

Structural inequalities impact the prevalence of mental health difficulties, receipt of support and outcomes from support. Some children, young people and families cannot afford to pay for activities that would improve their mental health. Indeed, children and young people with mental health difficulties are less likely to be able to afford to take part in out of school or college activities and recreational activities than those without mental health difficulties.^1^ Moreover, it is estimated that only a quarter of young people experiencing mental health difficulties receive specialist mental health support,^18^ and that over a quarter of referrals to specialist CAMHS are rejected.^19^ There is also high variation in waiting times, with one study analysing administrative data reporting that 47% of the sample waited 5–18 weeks following referral.^12^

A recent systematic review identified a need for public mental health interventions to improve knowledge of mental health problems, increase understanding and expectations of support and reduce stigma to improve young people’s mental health help-seeking.^20^ Young people repeatedly say that a key barrier to engaging with adults in the system around them, from schools to specialist services, is feeling judged and feeling that they are not taken seriously.^20^ This is exacerbated for young people from already marginalised groups.^21^ Thus, it is vital that young people are respected and have their voices heard. All young people have a legal right to be involved in decisions that affect them under the UN Convention on the Rights of the Child.^22^ Preventative personalised care is a central priority to health and social care, as is actively involving young people in their mental health support.^23 24^ Personalised care is directly supported by the National Institute for Health and Care Excellence (NICE), such as through shared decision-making.^25^ A person-centred perspective is also central to the research, recognising individuality and heterogeneous identities. A universally available public mental health intervention for young women that they can personalise based on their identities, making it inclusive of young women from marginalised groups, is needed to improve mental health and well-being at a population-level.

My Story and Me is a new public mental health intervention, specifically a digital storytelling intervention for young women and girls that was developed to help reduce stigma, improve mental health literacy and support early help-seeking. A logic model outlining the intervention components, mechanisms, outcomes and moderators is shown in online supplemental figure 1. My Story and Me provides a free, voluntary route for early intervention or prevention support which young people can access flexibly when and where they wish. It was designed to be inclusive of young women from marginalised groups, such as minoritised ethnic, LGBTQIA+ and neurodivergent groups, addressing a gap among existing interventions.^26^ My Story and Me builds on a previous study, in which we co-adapted (with permission) a widely used evidence-based intervention to personalised healthcare, ‘Ask 3 Questions’.^27^ This involved interviewing young women about their life experiences and asking them to share three stories: Who am I?, What is my mental health story?, How would I like to be supported? These interviews were turned into short stories providing rich descriptions of their identities, backgrounds and mental health journeys, and how these impacted each other. The recorded stories form a library on the My Story and Me digital platform, available for users to view (see Procedures section for further details).

Digital storytelling interventions can be categorised as educational, skill-building, enabling understanding of other people’s lived experiences and enabling understanding of one’s own lived experiences.^28^ Systematic reviews of digital storytelling mental health interventions for young people identified that interventions were mostly educational or documentary-based, but none were similar to My Story and Me.^29 30^ A systematic review of digital storytelling interventions across health concluded that they are an empowering approach that honours culturally relevant information.^31^ Similarly, a recent systematic review of film-based interventions to improve mental health education for young people found evidence of promise for reducing stigma and improving attitudes towards help-seeking.^29^ This included three randomised controlled trials (RCTs): one in Portugal^32^ and one in Spain,^33^ which found that a film and group discussion intervention was superior to no-intervention in reducing stigma; however, no effects were found in one RCT with Arab American students.^34^

The primary aim of this study is to determine the optimum approach to implementing a future full trial of My Story and Me. The specific objectives are to (1) determine the most appropriate setting in which to deliver the intervention and conduct the full trial, (2) refine the sample size calculation for the full trial, (3) understand what constitutes a meaningful difference in the primary outcome (eg, anxiety and depression scores), (4) explore the acceptability of the intervention and the study, and (5) carry out feasibility work to inform a future economic evaluation.

## Methods and analysis

### Study design

The study is an 18-month, mixed-methods, uncontrolled feasibility study. The study start and end dates are March 2025 to September 2026, with data collection taking place between October 2025 and June 2026.

### Study setting

The study will be set in up to three secondary schools, three further education (FE) colleges and three community organisations in the UK. These settings will be selected to enable the feasibility testing of the study across diverse contexts with differing operational and engagement challenges. These settings have a wide reach of diverse populations of young women and girls aged 14–18 years old.

### Participants

Overall, up to 180 young women and girls (20 per site) aged 14–18 years and up to 36 staff members will be recruited with support from site leads.

#### Eligibility criteria

Inclusion criteria include identifying as a young woman or girl (see inclusive definition below), aged 14–18 years; no active suicidal ideation (identified through use of a screening questionnaire); not currently receiving or seeking specialist mental health support (defined as CAMHS or comparable support for severe mental health difficulties); proficient in English (written and verbal), and able to provide informed consent (with parent/carer consent if aged under 16 years).

We use the terms ‘girl’ and ‘young woman’ inclusively. If potential participants are gender diverse and feel that this topic is relevant to them, then we welcome their views. We will respect participants’ gender.

#### Site recruitment

Sites will be recruited through our networks and those of the partner organisations, including websites, newsletters, social media channels and online searches. Interested sites will complete an online site characteristics survey, including a breakdown of young people’s sociodemographic characteristics and special educational needs. We will intentionally select sites that are more diverse to maximise recruiting participants from marginalised backgrounds.

The site characteristics survey will also ask about existing mental health and well-being support. Sites will be excluded if existing support is similar to My Story and Me (ie, digital storytelling intervention) or if there has been a serious incident in the past 12 months that would make participating in the study inappropriate or unfeasible. A serious incident may include experiences which are traumatic and/or affect the whole community of the site, such as deaths or injury of a peer or members of the community or harm to others. In this context, My Story and Me may not be the right level of support for young people. We also want to make sure that participants are not adversely affected by any of the content on the platform.

Eligibility will be determined after a call between the site and the research team. We anticipate several consultations with each site to coproduce plans for participant recruitment and delivering the study.

#### Participant recruitment

Potential participants will be recruited via school or college assemblies, group meetings, posters/flyers and/or newsletters, depending on the site. Each site will create a recruitment strategy that involves introducing the study via a recruitment video followed by a question-and-answer session with the research team. Everyone will receive an information sheet, either digitally or as a hard copy, which includes a link to the expression of interest (EOI) form. Participation is fully voluntary.

The link will guide interested young people aged 16–18 years directly to the EOI form, consent form and screening questionnaire. Those aged under 16 years can express interest through the EOI form, and then the sites will contact their parents/carers and share the information sheets and consent forms for them and young people to complete. The screening questionnaire includes the inclusion criteria to determine participant eligibility. Young people will be informed by the research team whether they are eligible for the study and whether they have been selected to take part. The safeguarding contact at the recruiting site will be informed of any individuals screened with active suicidal ideation.

Sites will receive reimbursement to cover administrative costs. Young people will receive a £15 shopping voucher for completing a baseline questionnaire and a £25 voucher for the follow-up questionnaire. Staff taking part in an interview will receive a £15 voucher and young people interviewed will receive a £30 voucher.

#### Sample size

The target sample size is six to nine sites and 20 participants per site, for a total of 120–180 participants. This sample is appropriate for assessing recruitment, retention, intervention delivery and data-collection processes, and for estimating key parameters needed for the future definitive cluster RCT. It also aims to meet the average cluster size requirements based on our original sample size calculation for the intended future trial based on the primary outcome being the Short Mood and Feelings Questionnaire (SMFQ) at the 7-month follow-up. The target range represents roughly 20–30% of the anticipated sample size for the full trial. The feasibility data will be used to refine assumptions for the definitive trial, including the intraclass correlation coefficient and expected attrition, and to confirm that the planned recruitment approach is workable at scale.

We will conduct interviews with 15–18 young women and girls in each of the three settings (ie, 45–54 in total). We will also interview 10–12 staff per setting (ie, 30–36 in total).

To examine whether different groups have different experiences of the platform, we have set recruitment targets for interviews with young women and girls based on ethnicity (shown in table 1) and our three priority groups: participants who are neurodivergent, LGBTQIA+ or experiencing digital poverty. Our recruitment target is 20% (or 9–11 participants) for each of the three priority groups. The interview sample size is intended to gain sufficient breadth and depth of experiences from different demographic groups.

**Table 1:** Recruitment targets for interviews based on ethnicity

Ethnic group	Population %	Target %	Target n
Asian	12	12	5–6
Black	6	10	5–6
Mixed race	5	10	5–6
Other	2	10	5–6
White: English, Welsh, Scottish, Northern Irish or British	69	48	20–24
White: Other	5	10	5–6

Population % was taken from the Census 2021 for females aged 14–18 years.^45^

As every 25% of baseline and interview samples are recruited, we will review demographic characteristics to ensure that we are reaching the target numbers. If we have more interest in participating in the study than our target sample size allows, we will prioritise those within our three priority groups.

### Procedures

Questionnaires will be completed by participants online with support from the research team and/or site staff either in person or virtually, depending on the site.

Interviews will be conducted by members of the research team, including the peer researcher. Interviews will be audio recorded using an encrypted dictaphone (or via Microsoft Teams) and transcribed by an external agency with which we have a data sharing agreement.

My Story and Me is a personalised public mental health intervention hosted on an online platform. It involves watching mental health stories created by young women from different ethnic, LGBTQIA+ and neurodivergent groups, including those from multiply marginalised groups. In these videos, girls and young women answer three questions: ‘Who am I? What is My Mental Health Story? How would I Like to be Supported?’. The intervention draws on the theory of mentalisation—that by learning how someone else understands their behaviour and mental states (ie, hearing the mental health stories of others with shared identities), young women will understand their own behaviours and mental states better.^35^

Participants will be asked to access the platform for how long or often they would like. Participants are encouraged to record their own story, and they may choose to answer the same three questions answered in the existing videos. All participants can create stories using text or audio, but only those 16 years and older can create a video story. Only the young woman creating their story and platform administrators have access to their story. Platform administrators will not access the stories; only a member of the research team would access a recording if a participant has disclosed that they have recorded something that raises a safeguarding concern to the research team or site staff.

Study participants will not be able to make their own story public during the study. At the end of the study, participants will have the option to release a story to the public platform. Before doing so, we will collect the necessary consent(s), and they will select a story (video/audio/text), which will be moderated before release.

#### Safeguarding

The platform has been carefully designed, with clinical psychologists, to minimise the risk that the content could cause distress. Each story has a description, a note flagging sensitive content, a summary of themes, a transcript, further information on the main topics (eg, bullying) and signposting to relevant helplines and resources. There will be limits on the number of videos a participant can watch in one sitting (five videos) and a ‘leave now’ button if they want to quickly exit the platform. The platform also includes general signposting to helplines and resources and specific guidance on how users can talk about their mental health with others (eg, teachers, doctors, community leaders).

Any safeguarding issues will be flagged to the principal investigator, their line manager or another senior member of the team, as well as a safeguarding contact at each participating school, FE college and community organisation. We will work with these contacts to collate specific signposting resources for participants from that site to use.

Participants will be informed in study information sheets that the research team will not routinely check private stories and that confidentiality of recorded stories will only be broken if the participant shares with a member of staff from the site or research team that they have recorded something that raises a safeguarding concern. In this case, the research team will review the story and follow safeguarding procedures if needed.

#### Adverse events protocol

Site staff will report adverse events for participating young people anonymously. These will be reviewed by an Adverse Events Oversight Group comprised of independent senior researchers and clinicians who will oversee decisions made about the study’s continuation.

### Measures

#### Quantitative measures

Participating young people will complete a survey at baseline and 7-month follow-up. A range of outcome measures will be used to determine the feasibility and impact of the intervention and inform the future economic evaluation (see online supplemental table 1). The questionnaires will take approximately 25 min to complete.

Additionally, we will capture young people’s activity on the digital platform to measure levels of engagement and adherence to the model (eg, young women will be classified as ‘adherent’ if they meet the threshold of watching one video and creating one story).

#### Qualitative measures

The research team will record a ‘site log’ to document barriers and facilitators to conducting the study in each site, both from their perspective and the perspectives of site staff. This will include details of onboarding the site, participant recruitment, implementing the intervention, data collection and use of anti-stigma guidance materials. It will also capture demographic characteristics of the site and types of well-being support offered.

Interviews will be conducted by the research team from approximately 6 weeks after baseline measures are completed. Topics explored will include participants’ views and experiences of My Story and Me; views of the study materials (ie, recruitment resources, information sheet, consent form and outcome measures); and engagement in the study; and changes experienced since using the platform, including potential meaningful changes for future participants. We will also ask for their views on future participants being allocated to different conditions within a future full trial and the acceptability of randomisation. Interview schedule development will be informed by the Young People’s Advisory Group (YPAG).

Staff from each setting will be interviewed to examine their experiences of delivering the intervention and research procedures. Across settings, we will examine differences in engagement, embedding of the platform into the everyday lives of young people and any barriers and facilitators to implementation. Treatment as usual (ie, other well-being support) will be discussed to compare across the three settings. We will also examine the use and impact of the anti-stigma materials provided. Interview schedule development will be informed by the newly updated consolidated framework for implementation research.^36^

### Progression criteria

Data collected from the site characteristics surveys, site logs, interviews, outcome measures and engagement and activity monitoring will be used to inform any changes to study procedures and potentially the intervention, as well as the decision to proceed with the intended full trial. A key consideration, particularly about the setting(s) in which to conduct the full trial, will be the resources required to deliver the study and the setting-specific progression criteria ratings (see table 2).

**Table 2:** Progression criteria for settings to proceed to full trial without moderation

Progression criteria	Rating
Recruitment	Green for 100% for both site (ie, 2) and young women and girls (ie, mean ≥20/site). Amber for ≥70% for young women and girls (ie, mean/site ≥14 but <20). Red for less than 100% for sites (ie, <2) or <70% for young women and girls (ie, mean/site <14).
Retention	Green for <30% loss to 7-month follow-up. Amber for 30–40% loss to 7-month follow-up. Red for >40% loss to 7-month follow-up.
Fidelity	Green for at least 75% of young people meeting the minimum level of intervention engagement (watch one story and create one story). Amber for <75% but ≥50%. Red for <50%.
Intervention acceptability	Green for at least 75% of young people rating My Story and Me as acceptable. Amber for <75% but ≥50%. Red for <50%.
Randomisation acceptability	Green for at least 75% of young people rating randomisation as acceptable. Amber for <75% but ≥50%. Red for <50%.
Safety	Green for ≤5% of participants (ie, 6) experience an adverse event related to the study. Amber for >5% but ≤10% of participants (ie, 6–12) experience an adverse event related to the study. Red for >10% of participants experience an adverse event related to the study or there is one serious adverse event related to the study (there will be a review about stopping the feasibility study if either red threshold is met).
Logic model	Green for no updates to the logic model needed or minimal changes required based on findings from the feasibility study. Amber for substantive changes required. Red for findings that rule the logic model null and void.

### Data analysis

#### Quantitative data analysis

The primary purpose of the quantitative analysis in this feasibility study is to inform decisions about the design and delivery of a future full trial of My Story and Me. Specifically, the analysis will assess the feasibility, acceptability and preliminary signals of potential impact across different settings (secondary schools, FE colleges and community organisations), with a focus on reaching young women and girls from marginalised groups. All quantitative analyses will be descriptive and exploratory in nature, in line with the feasibility study aims.

To examine whether different settings enable access to young women and girls with varying levels of mental health need, we will compare the demographic characteristics and baseline scores on outcome measures across the three setting types. Particular attention will be paid to representation of priority groups (eg, participants who identify as LGBTQIA+, are neurodivergent or experience digital poverty). The Revised Children's Anxiety and Depression Scale (RCADS) will be used to assess differences in baseline symptoms of depression and anxiety between settings, alongside other validated measures including the SMFQ, Generalised Anxiety Disorder 7 and Reflective Functioning Questionnaire. These comparisons will allow us to evaluate whether different settings reach young people with differing levels of need and will inform the selection of settings for the full trial.

Platform-level engagement data will be used to assess fidelity and adherence to the intervention. We will examine the extent to which young people used the My Story and Me platform, including the number of stories watched, story formats chosen and whether they created a personal story. A minimum threshold for adherence will be defined as watching at least one video story and creating one personal story (text, audio or video). We will explore differences in engagement by setting and by key sociodemographic characteristics, particularly private digital access, to assess whether digital exclusion affects platform use.

Descriptive statistics will be used to summarise baseline and follow-up data. For continuous variables, means, medians and SD will be reported; for categorical variables, frequencies and percentages will be presented. These data will be used to update key parameters for the full trial, including estimates of cluster size, recruitment rates per site, retention rates and variability in primary and secondary outcomes. Subgroup analyses will be conducted to explore whether participation rates and baseline mental health needs vary across settings and among different sociodemographic groups. χ^2^ or Fisher’s exact tests will be used for categorical comparisons, and t-tests or non-parametric equivalents (as appropriate) will be used for continuous variables. The main aim of collecting follow-up data is to assess completion rates. Pre–post changes in primary and secondary outcomes will be explored, although these will be underpowered.

This study includes prespecified progression criteria across multiple domains (eg, recruitment, retention, fidelity, acceptability and safety). The decision to proceed to a full trial will be informed by these criteria. Progression will not be recommended if recruitment rates suggest that more than 50 sites would be needed to meet sample size requirements for the full trial. It will also not be recommended if two or more progression criteria are rated red or if the Study Steering Committee does not approve remediation plans for amber or red criteria, including strategies to improve adherence.

#### Qualitative data analysis

Drawing on the framework analysis approach,^37^ interview data will be grouped by setting and different identities (eg, ethnicity, LGBTQIA+, neurodivergent) to enable differences in views, experiences and change processes to be examined across settings and for different sociodemographic groups. Initially, interview data will be analysed deductively, using predefined categories relating to our research questions within which to organise and code the interview data. Category development will also be informed by the CFIR.

Interview data coded or assigned to these deductively identified categories will then be analysed inductively within these categories, drawing on a reflexive thematic analysis approach.^38 39^ Researchers will independently code each transcript using the NVivo qualitative data analysis software package, with oversight from the qualitative research lead. Researchers will meet regularly with each other and the wider study team to discuss codes and develop a provisional thematic framework. The final thematic framework will be presented to the study team and YPAG, who will help to ensure that it remains grounded in the data and that it reflects participants’ lived experiences. Analysis will occur in parallel to data collection. We shall use the qualitative findings to particularly interrogate theorised impact and the pathways to impact outlined in our logic model, as well as barriers and facilitators to implementation.

We will report descriptively on treatment as usual. This will be complemented by young women and girls reporting what support they have received in and outside of the setting. This will enable us to examine the complexity and variability of treatment as usual and similarities and differences between the three settings.

#### Site logs

Narrative summaries will be produced from the site logs for each of the three settings, including a description of the similarities and differences in the research processes within each setting. An overarching narrative summary will be produced from the three setting-level narrative summaries, which will then be used to inform decisions about the intended future trial.

#### Data synthesis

Findings across the different strands of data collection—site characteristics surveys, site log narrative summaries, interviews, outcome measures and engagement and activity monitoring—will be synthesised to inform progression criteria and the overall logic model for the intervention.

### Governance and oversight

The study will be overseen by a Study Steering Committee, Data Monitoring and Ethics Committee and Adverse Events Oversight Group. Quarterly Co-Investigator meetings and a YPAG will support delivery and equity monitoring throughout the study.

### Data protection

A data protection impact assessment has been completed and agreed by the Data Protection Officer at Anna Freud. Participants will receive a privacy notice outlining their data protection rights alongside the study information sheet, in line with General Data Protection Regulation regulations.

All data will be collected and managed on secure systems. Only approved researchers will have access to the data. Only pseudonymised survey responses and transcripts of discussions will be shared with the partner organisations (eg, Kings College London) so that they can help us to analyse them. The information will only be shared securely, and we will make sure that they protect the information. Data will only be accessed following signing of the Collaboration Agreement and Data Sharing Agreement. At the end of the project, anonymised data will be archived and retained for at least 10 years, at which point there will be a data retention review.

### Study registration

This study was registered with ISRCTN (ISRCTN12191423) on 22 May 2025.

### Equality impact assessment

We have conducted an Equality Impact Assessment^40^ informed by National Institute for Health and Care Research guidance (eg,^41 42^). This study seeks to address mental health inequalities faced by girls and young women by testing an intervention that hopes to tackle the stigma associated with mental ill-health and help-seeking, particularly for those facing marginalisation or who hold multiple and intersecting identities. Through the assessment, we have identified parts of the research and/or intervention that are less accessible to certain groups of people. For example, because the intervention is in English, language may be a barrier to participation. We will work with sites to help reduce this barrier, monitor how many potential participants are being excluded due to language and recommend including stories in other languages in the future. Other actions arising from this assessment include recruiting sites with diverse populations, creating anti-stigma guidance for sites to use during the study, enabling accessibility features in the digital platform (eg, subtitles and transcripts), and monitoring digital access for participants.

### Patient and public involvement

The patient and public involvement (PPI) within the feasibility study aims to embed youth voice throughout the project, using the Lundy model principles of space, voice, audience and influence to ensure that My Story and Me is effective and relevant to young people.^43^ Diverse populations were sought for the PPI in this project to reflect the priority groups outlined above, namely groups marginalised due to ethnicity, sexual orientation, gender identity, and/or neurodiversity.

First, we recruited a Platform Development Group comprised of young women and girls from diverse groups with lived experience of mental health difficulties. The aim of this group was to provide insight and feedback on the digital platform prior to and throughout its development over three workshops. This included feedback on the user interface, order of pages on the site, processes and user experience, colours and the language used on the platform. The feedback helped to ensure the platform is relevant, relatable, accessible, and sensitive to a range of young people.

A YPAG has also been created to ensure that we embed youth voice throughout all stages of the project, including study preparation, recruitment, data collection and analysis. Diverse young women and girls from our priority groups were recruited, and members may also have lived experience of mental health difficulties. The YPAG will review participant-facing recruitment materials, review and test outcomes measures, help develop study material (such as the young person recruitment video) and help interpret the findings from interviews. The YPAG will meet about every 3 months with other ad-hoc tasks completed over email. The YPAG will be reimbursed with vouchers of £25 per person per 1.5-hour session to compensate for their time and input; additional activities will be reimbursed at the same rate.

Lastly, the Peer Researcher, who has been present throughout the development of the project, was appointed so that a young woman with lived experience relevant to the study is firmly embedded within the research team. They will oversee and advise on the study at a strategic and delivery level and undertake data collection and analysis activities as part of the qualitative study to strengthen the insights gathered. Additionally, they will work alongside research colleagues to develop other areas of PPI such as the planning and delivery of YPAG sessions.

## Ethics and dissemination

Ethical approval has been obtained from the University College London Research Ethics Committee, approval number 0692. Interested participants will be required to complete an expression of interest and consent form to take part in the study, and young people under 16 years old will be required to obtain parent/carer informed consent.

We will disseminate research findings using different outputs for different audiences. To disseminate findings to researchers, two open access peer-reviewed publications will be produced, including lived experience summaries: protocol and feasibility study findings. Early career researchers on the project will also attend one academic conference. A briefing on key messages and findings will also be distributed to local and national policymakers, drawing on existing networks (eg, Mental Health Policy Research Unit, Child Policy Research Unit, Department for Education, Department for Health and Social Care, Youth Justice Board). Finally, our plans for the full trial include identifying spokespeople from different communities (eg, influencers, leaders) to champion the project.

## Discussion

This non-randomised uncontrolled mixed-methods feasibility study aims to inform the design and implementation of a future full trial of My Story and Me. Specifically, it will help us identify the most suitable trial setting, refine the parameters for sample size calculations, determine a meaningful change in the primary outcome measure, assess the acceptability of the intervention and future trial, and determine the requirements for an economic evaluation.

The intended future trial will investigate whether My Story and Me plus treatment as usual (TAU) is more effective at reducing depression and anxiety for young women than TAU alone. This will be tested through a pragmatic cluster randomised controlled superiority trial with economic evaluation.

### Limitations, risks and practical challenges

We anticipate unique challenges for recruitment and delivery for the different settings. Engaging secondary schools will require time and resources that compete with other school priorities and staff’s heavy workloads. Recruiting from FE colleges may also be complex due to having a less structured environment compared with secondary schools (eg, individualised timetables), which may require multiple communication strategies. Similarly, community organisations will need to tailor their recruitment strategy depending on the type of contact they have with young people (virtual vs in-person).

As My Story and Me is a self-directed intervention with no prescribed usage, there may be a challenge to maintain user engagement with the platform and subsequently the 7-month follow-up surveys. Mitigation strategies include sending young people regular study updates and/or notifications from the platform and offering tiered reimbursements for completing surveys and interviews.

Given the platform’s digital nature, we will monitor and address digital access and usability challenges, particularly for participants with limited digital literacy or access. As part of the baseline and follow-up measures, we will ask participants about their levels of private digital access. In the analysis, we will examine if there are differential levels of engagement and interaction with My Story and Me—using the activity data recorded in the platform—for young women and girls who report lower levels of private digital access. We will examine recruitment rates to examine differences between those with and without private digital access. This will enable us to examine the potential need for providing participants with devices in the full trial.

We also recognise the limitation of offering the intervention in English only and will work with sites to try to reduce barriers to participation that are due to language. We hope to adapt My Story and Me to some of the more widely spoken secondary languages in the UK in the future.

Finally, the single-arm study design limits the ability to fully examine the acceptability of randomisation for a future trial. This limitation will be mitigated through qualitative interviews with participants to explore their views on being allocated to different conditions.

### Strengths of the study or study design

A feasibility study is an important learning phase that allows us to trial the intervention in different settings and uncover practical challenges, which will enable us to refine the intervention and methods before investing in a large-scale trial.

The intervention My Story and Me addresses several gaps in the literature. Specifically, there is a lack of RCTs evaluating video-based public mental health interventions for young people in the UK. There is also a gap in interventions that enable young people to build their own mental health stories, and especially interventions that are tailored to multiple marginalised groups, enabling application at a whole population-level. This feasibility study is the first step to adding to the evidence base on digital public mental health interventions for a wide range of young women and girls in the UK. This will in turn support management of the growing rate since 2017 of young women between the age of 17 and 25 experiencing mental health difficulties^1^; due to the disproportionate impact of the COVID-19 pandemic^9 10^ and the increasing sexual harassment that up to 90% of young women have been experiencing.^8^

A core strength of the study is the integration of youth voice throughout the study, following the Lundy model principles for PPI. From the development stage, the intervention has been designed with feedback from young women and girls. We will continue to be guided throughout the study by a YPAG, made up of a diverse group of young women and our peer researcher to ensure that girls and young women with lived experience shape our research and are part of our decision-making.

Our recruitment strategies are focused on ensuring that the research is inclusive and representative of seldom heard or marginalised groups of young people. We will purposively sample sites to reflect sociodemographic diversity and ensure a spread in ethnic backgrounds and special educational needs. In our participant recruitment materials, we have an inclusive definition of young women and girls (defined earlier) and encourage participation from young people from the LGTQIA+ community, neurodivergent individuals and people from minoritised ethnic groups.

Finally, by producing these findings informing the ultimate output of the project, the launch of the My Story and Me platform, we aim to empower young women and girls to tell their stories on their own terms and seek support earlier. To this end, it is intended that My Story and Me will be a low cost, sustainable, scalable and inclusive intervention with the potential to reduce the high societal cost of mental illness (during youth and adulthood) for young women from marginalised and multiply marginalised groups.^44^

## Supplementary material

10.1136/bmjopen-2025-115245 online supplemental figure 1

10.1136/bmjopen-2025-115245 online supplemental table 1
