# Supplementary figures and images for: My Story and Me: protocol for a feasibility study of a personalised public mental health intervention for young women aged 14–18 years
Source: BMJ Open. 2026 May 6;16(5):e115245. doi: 10.1136/bmjopen-2025-115245 (PMC13150907; doi:10.1136/bmjopen-2025-115245)

**Supplementary Figure 1.** Logic model of the intervention

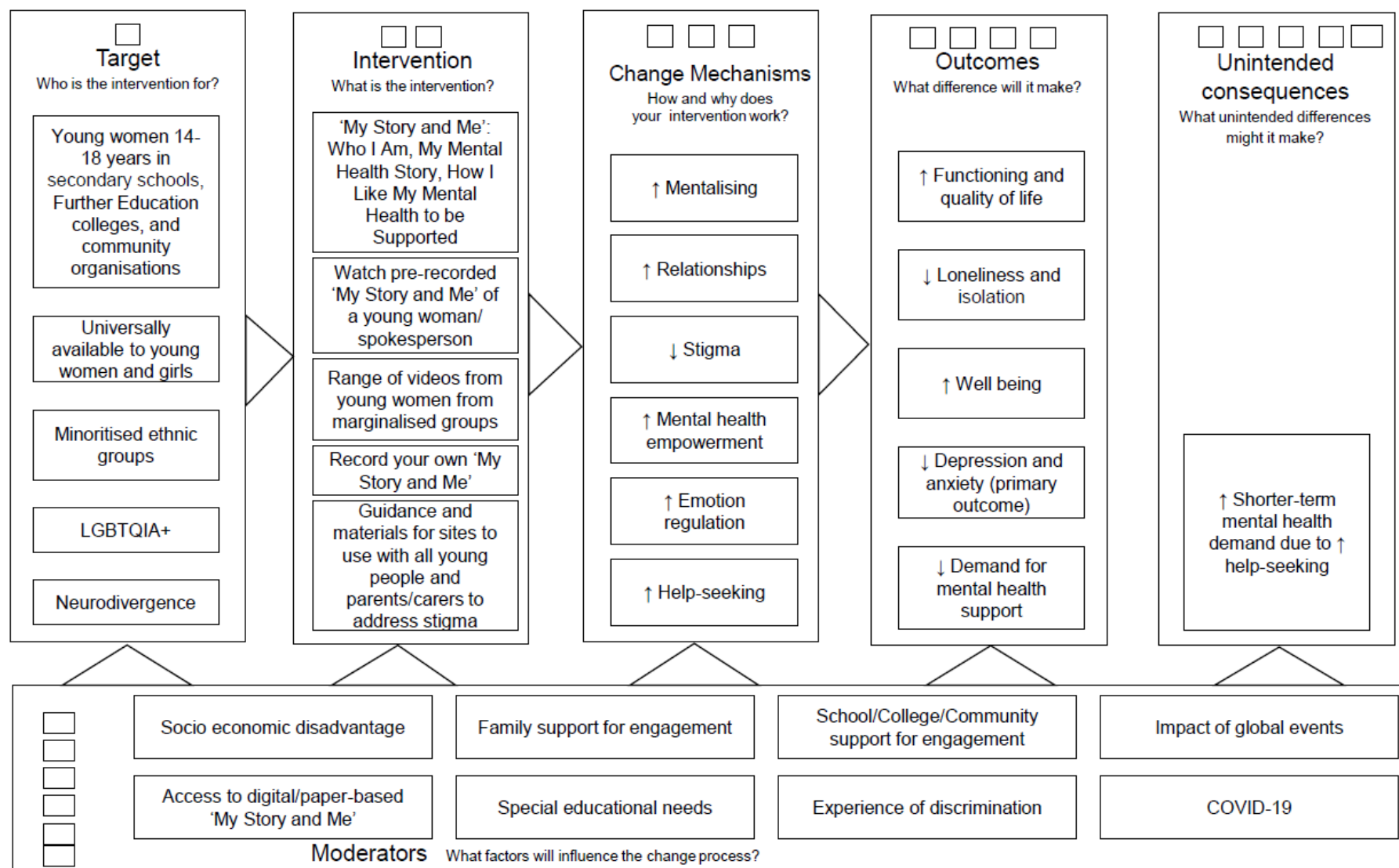

Supplement: online supplemental figure 1 [file bmjopen-16-5-s001.pdf]
