# Supplementary material for: My Story and Me: protocol for a feasibility study of a personalised public mental health intervention for young women aged 14–18 years
Source: BMJ Open. 2026 May 6;16(5):e115245. doi: 10.1136/bmjopen-2025-115245 (PMC13150907; doi:10.1136/bmjopen-2025-115245)
Supplement: online supplemental table 1 [file bmjopen-16-5-s002.pdf]

Supplementary Table 1. *Outcome measures for the feasibility study*

**Primary Outcome (validated measure)**		
**Outcome**	**Measure**	**Time point**
**Depression and anxiety symptoms**	Short Mood and Feelings Questionnaire (SMFQ)<br><br>SMFQ is recommended in NICE guidelines for identifying depression in young people [1]. It is a widely used measure with evidence of validity and reliability [2], it has been used in previous similar trials [3], and it has been used with neurodivergent populations [4]. The PPI Lead/ Peer Researcher and Young People's Advisory Group (YPAG) have reviewed the measure and deemed it appropriate for use with neurodivergent participants in this trial.	Baseline and 7-month follow up
**Secondary Outcome (validated measures)**		

Outcome	Measure	Time point
**Anxiety**	Generalized Anxiety Disorder-7 (GAD-7)<br><br>This is a widely used measure with evidence of reliability and validity for the age range of this feasibility study [5].	Baseline and 7-month follow up
**Depression and anxiety**	11-item Revised Children's Anxiety and Depression Scale (RCADS)<br><br>This is a widely used measure with evidence of validity and reliability for young people [6].	Baseline and 7-month follow up
**Social support**	12-item Multidimensional Scale of Perceived Social Support (MSPSS)<br><br>This is a widely used measure with evidence of validity and reliability for young people [7], and it has been with neurodivergent	Baseline and 7-month follow up

	young people in NIHR funded trials [8].	
**Mentalising**	5-item Reflective Functioning Questionnaire Youth-5<br><br>This is a widely used measure with evidence of validity and reliability for young people [9]. The PPI Lead/ Peer Researcher and YPAG have reviewed the measure and deemed it appropriate for use with neurodivergent participants in this trial. They helpfully suggested wording to introduce the measure to help increase accessibility.	Baseline and 7-month follow up
**Stigma**	6-item Mental Health Knowledge Schedule<br><br>This is a widely used measure with evidence of validity and reliability [10] and has been used in	Baseline and 7-month follow up

	previous school-based mental health trials [11].	
**Quality of life**	6-item EQ-5D-5L [12] and the 9-item CHU-9D [13]	Baseline and 7-month follow up

Secondary outcomes and assessments (non-validated measures)

Outcome	Measure	Time point
**Loneliness**	Single item on loneliness [14]<br><br>This was chosen to reduce measurement burden.	Baseline and 7-month follow up
**Mental health empowerment**	4 items<br><br>These are part of a measure developed by the research team.	Baseline and 7-month follow up
**Intervention acceptability**	7-item Theoretical Framework of Acceptability [15]	7-month follow up
**Resource use**	An adapted version of the Child and Adolescent Service Use Schedule [16].	Baseline and 7-month follow up

	The PPI Lead/ Peer Researcher and YPAG have reviewed the measure and suggested amendments, including the measure being shorter and condensed, utilising less formal language, and providing definitions and further explanations for some services (e.g., respite care - places to let you have a break temporarily from the person you usually care for).	
**Randomisation acceptability**	Single item on randomisation acceptability	Baseline
**Demographic characteristics**	Age, gender, sexual orientation, ethnicity, religion, special educational needs, disability, care experience, and caring responsibilities; postcode of residence will be collected and mapped onto national data for area-level indices of deprivation	Baseline

Supplementary References

1. National Collaborating Centre for Mental Health (UK). Depression in children and young people: identification and management in primary, community and secondary care. British Psychological Society (UK); 2005.

2. Thabrew H, Stasiak K, Bavin LM, et al. Validation of the Mood and Feelings Questionnaire (MFQ) and Short Mood and Feelings Questionnaire (SMFQ) in New Zealand help-seeking adolescents. Int J Methods Psychiatr Res. 2018;27(3). https://doi.org/10.1002/mpr.1610

3. Pile V, Smith P, Leamy M, et al. A feasibility randomised controlled trial of a brief early intervention for adolescent depression that targets emotional mental images and memory specificity (IMAGINE). Behav Res Ther. 2021;143. https://doi.org/10.1016/j.brat.2021.103876

4. Rai D, Culpin I, Heuvelman H, et al. Association of autistic traits with depression from childhood to age 18 years. JAMA Psychiatry. 2018;75(8). https://doi.org/10.1001/jamapsychiatry.2018.1323

5. Tiirikainen K, Haravuori H, Ranta K, et al. Psychometric properties of the 7-item Generalized Anxiety Disorder Scale (GAD-7) in a large representative sample of Finnish adolescents. Psychiatry Res. 2019;272. https://doi.org/10.1016/j.psychres.2018.12.004

6. Radez J, Waite P, Chorpita B, et al. Using the 11-item Version of the RCADS to Identify Anxiety and Depressive Disorders in Adolescents. Res Child Adolesc Psychopathol. 2021;49(9). https://doi.org/10.1007/s10802-021-00817-w

7. Bruwer B, Emsley R, Kidd M, et al. Psychometric properties of the Multidimensional Scale of Perceived Social Support in youth. Compr Psychiatry. 2008;49(2). https://doi.org/10.1016/j.comppsych.2007.09.002

8. Wright B, Kingsley E, Cooper C, et al. I-SOCIALISE: Results from a cluster randomised controlled trial investigating the social competence and isolation of children with autism taking part in LEGO® based therapy (‘Play Brick Therapy’) clubs in school environments. Autism. 2023;27(8). https://doi.org/10.1177/13623613231159699

9. Sharp C, Steinberg L, McLaren V, et al. Refinement of the Reflective Function Questionnaire for Youth (RFQY) Scale B Using Item Response Theory. Assessment. 2022;29(6). https://doi.org/10.1177/10731911211003971

10. Evans-Lacko S, Little K, Meltzer H, et al. Development and psychometric properties of the mental health knowledge schedule. Can J Psychiatry. 2010;55(7). https://doi.org/10.1177/070674371005500707

11. Hayes D, Moore A, Stapley E, et al. Promoting mental health and wellbeing in schools: examining Mindfulness, Relaxation and Strategies for Safety and Wellbeing in English primary and secondary schools: study protocol for a multi-school, cluster randomised controlled trial (INSPIRE). Trials. 2019;20(1). https://doi.org/10.1186/s13063-019-3762-0

12. Herdman M, Gudex C, Lloyd A, et al. Development and preliminary testing of the new five-level version of EQ-5D (EQ-5D-5L). Qual Life Res. 2011;20(10). https://doi.org/10.1007/s11136-011-9903-x

13. Stevens K. Valuation of the child health utility 9D index. PharmacoEconomics. 2012;30(8). https://doi.org/10.2165/11599120-000000000-00000

14. ONS. Measuring loneliness: guidance for surveys [online]. 2018. https://www.ons.gov.uk/peoplepopulationandcommunity/wellbeing/methodologies/measuringlonelinessguidanceforuseofthenationalindicatorsonsurveys

15. Sekhon M, Cartwright M, Francis JJ. Development of a theory-informed questionnaire to assess acceptability of healthcare interventions. BMC Health Serv Res. 2022;22(1). https://doi.org/10.1186/s12913-022-07577-3

16. Byford S, Barrett B, Roberts C, et al. Cost-effectiveness of selective serotonin reuptake inhibitors and routine specialist care with and without cognitive-behavioural therapy in adolescents with major depression. Br J Psychiatry. 2007;191. https://doi.org/10.1192/bjp.bp.107.038984
