# Supplementary material for: My Story and Me: protocol for a feasibility study of a personalised public mental health intervention for young women aged 14–18 years
Source: BMJ Open. 2026 May 6;16(5):e115245. doi: 10.1136/bmjopen-2025-115245 (PMC13150907; doi:10.1136/bmjopen-2025-115245)
Supplement: Supplementary file 3 [file bmjopen-2025-115245.reviewer_comments.pdf]

PEER REVIEW HISTORY

BMJ Open publishes all reviews undertaken for accepted manuscripts. Reviewers are asked to complete a checklist review form and are provided with free text boxes to elaborate on their assessment. These free text comments are reproduced below.

ARTICLE DETAILS

Title (Provisional)

My Story and Me: protocol for a feasibility study of a personalised public mental health intervention for young women aged 14 to 18 years

Authors

Labno, Angelika; Abel, Kathryn Mary; Aked, Hil; Bhui, Kamaldeep; Brooks, Helen; Crease, Maddison; Deighton, Jessica; Edridge, Chloe; Fonagy, Peter; Grace, Eleanor; Heslin, Margaret; Leyera, Suzet Tanya; Nisbet, K.; Obateru, Adetola; Norton, Sam; Ravaccia, Giulia; Stapley, Emily; Edbrooke-Childs, Julian

---

VERSION 1 - REVIEW

**Reviewer**	**1**
**Name**	**Nollett, Claire**
**Affiliation**	**Cardiff University, Centre for Trials Research**
**Date**	**03-Mar-2026**
**COI**	**None**

This is a protocol paper looking at the feasibility and acceptability of an online intervention for girls and young women with anxiety and depression delivered via schools and colleges. The methods are well described and the outcomes are largely appropriate for a feasibility study. I have a few suggestions/clarification requests for the authors:

1) In abstract line 24 word 'with' is missing after qualitative interviews

2) In strengths and limitations of this study - you have said that a limitation is the uncontrolled design which limits interpretation of the intervention's effectiveness and ability to make causal inference - however, I would argue that as this is a feasibility study, this is not the aim of the study and hence, this isn't a limitation. What is a limitation of a single arm study is ability to thoroughly assess acceptability of randomisation.

3) I would have liked to see more information on how My Story and Me was developed (how was it developed from Ask 3 Questions and why) and perhaps a TiDier checklist would help with explaining the intervention, or a theory of change or logic model. How do the parts of the intervention act on the intended outcomes?

4) Procedures - What is the rationale for study participants not being able to make their own story private during the study (if making and sharing a story is part of the intervention)?

5) Qualitative measures - 'we will ask for their views on future participants being allocated to different conditions within a future trial' - would the future trial not be a cluster randomised trial (to avoid contamination) without individual randomisation?

VERSION 1 - AUTHOR RESPONSE

Reviewer comments

In abstract line 24 word 'with' is missing after qualitative interviews.	This has been addressed.
In strengths and limitations of this study - you have said that a limitation is the uncontrolled design which limits interpretation of the intervention's effectiveness and ability to make causal inference - however, I would argue that as this is a feasibility study, this is not the aim of the study and hence, this isn't a limitation. What is a limitation of a single arm study is ability to thoroughly assess acceptability of randomisation.	Revised the bullet point to: *"The single-arm study design limits the ability to fully examine the acceptability of randomisation."*<br><br>Also, we have expanded this point under Limitations, Risks, and Practical challenges: *"Finally, the single-arm study design limits the ability to fully examine the acceptability of randomisation for a future trial. This limitation will be mitigated through qualitative interviews with participants to explore their views on being allocated to different conditions."*
I would have liked to see more information on how My Story and Me was developed (how was it developed from Ask 3 Questions and why) and perhaps a TiDier checklist would help with explaining the intervention, or a theory of change or logic model. How do the parts of the intervention act on the intended outcomes?	We have included a logic model in the supplementary materials of the manuscript, which is now referenced in the introduction: *"A logic model outlining the intervention components, mechanisms, outcomes, and moderators is shown in Supplementary Figure 1."*<br><br>We do not include details on how the different components of the intervention affect outcomes because we aim to explore and test this through the feasibility study.<br><br>To give you more information on how My Story and Me was developed from Ask 3 Questions, My Story and Me adopted the structured prompting found in Ask 3 Questions. We co-created three fit-for-purpose questions intended to facilitate reflection and produce a mental health narrative. In a separate study, we recruited young women and girls to produce their mental health narratives for the story library

	on the digital platform. The intervention involves watching these young women's narratives, creating one's own narrative, and accessing mental health guidance and resources.<br><br>To reduce replication, we have clarified information in the introduction explaining the intervention development and included signposting to the Procedures section for further details on the intervention:<br>*"These interviews were turned into short stories providing rich descriptions of their identities, backgrounds, and mental health journeys, and how these impacted each other. The recorded stories form a library on the My Story and Me digital platform, available for users to view (see Procedures section for further details).*
Procedures - What is the rationale for study participants not being able to make their own story published during the study (if making and sharing a story is part of the intervention)?	Thank you for your helpful question. While creating your story is part of the intervention, sharing it on the platform is not. Instead, there will be guidance available on the platform on how participants can share their story with a trusted adult (e.g., relatives, health professionals, teachers). This is one of the ways in which we hope to encourage young people to seek help earlier.<br><br>In the future (e.g., the full trial), the platform may expand to give participants the option to add their story to a public library (subject to participant consent, a moderation process, and approval).
Qualitative measures - 'we will ask for their views on future participants being allocated to different conditions within a future trial' - would the future trial not be a cluster randomised trial (to avoid contamination) without individual randomisation?	You are correct in understanding that the future trial would be a cluster randomised trial. To clarify, the interview question will refer to allocation to different study conditions rather than individual randomisation (e.g., *"In a future study, people may be placed into different groups, where some people receive the intervention and others receive something else. What do you think about this?"*).

Additional changes

	We added the following acknowledgement: "We thank Emily McDougal for a review of the qualitative analysis."
	We were not able to input several authors' ORCID into the system, as it would not allow

	us to edit this part of their author details.<br><br>If you can manually input these, please include:<br><br>Chloe Edridge: 0000-0001-6697-3225<br>Emily Stapley: 0000-0003-2935-4438<br>Jessica Deighton: 0000-0002-9516-7545<br>Suzet Tanya Lereya: 0000-0001-9100-459X<br>Helen Brooks: 0000-0002-2157-0200<br>Kathryn M. Abel: 0000-0003-3538-8896<br>Sam Norton: 0000-0003-1714-9963

VERSION 2 - REVIEW

**Reviewer**	**1**
**Name**	**Nollett, Claire**
**Affiliation**	**Cardiff University, Centre for Trials Research**
**Date**	**15-Apr-2026**
**COI**	

Thank you for considering the points raised and making the relevant amendments to the manuscript.
